# 

**Published:** 1879-08

**Authors:** 


### CONTENTS.

COMMUNICATIONS.

Anæsthesia and Anæsthetics. *By Dr. J. J. Caldwell, of Baltimore, Md.* 317
A Review and Criticism on the Transactions of the Dental Association of Maryland and District of Columbia for the year 1878. *By Martin P. Scott, M. D., Baltimore, Md*............ 343

MISCELLANEOUS.

The Various Applications of Calcium Phosphate in Medicine.......... 348
The Hygrometric Properties of Glycerine.......... 349
Brain Work and Skull Growth.......... 351
Symes on Thymol and Thymol Camphor.......... 351
The Effects of Tobacco.......... 352
The Indiana Medical Protection Law.......... 353
The Medical Profession in Rome.......... 354

EDITORIAL.

The Siamese Teeth.......... 355
Transplanting.......... 356
The Results of Legislation.......... 357
Obituary.......... 358
Bibliographical.......... 359
American Health Primers.......... 360

